# Innate Lymphoid Cells: New Players in IL-17-Mediated Antifungal Immunity

**DOI:** 10.1371/journal.ppat.1003763

**Published:** 2013-12-12

**Authors:** André Gladiator, Salomé LeibundGut-Landmann

**Affiliations:** Institute of Microbiology, ETH Zurich, Zürich, Switzerland; The University of North Carolina at Chapel Hill, United States of America

## How Does IL-17 Mediate Antifungal Immunity?

The IL-17 cytokine family has emerged as a central component of the host immune system since its discovery two decades ago. IL-17A and IL-17F, the two signature cytokines collectively referred to as IL-17 in the following, have attracted much attention owing to their pathological potential and their ability to promote autoimmune diseases such as psoriasis and rheumatoid arthritis. It has become clear however that IL-17 also plays a key role in host protection from infection with extracellular bacteria and fungi. IL-17A and IL-17F homo- and heterodimers bind to a heterodimeric complex composed of IL-17RA and RC receptor subunits and trigger a signaling cascade that results in the activation of NF-κB and MAPK [1]. This results in the production of antimicrobial compounds and inflammatory mediators including G-CSF and CXC chemokines, which are believed to be the primary basis for recruitment and activation of neutrophils [1]. Recently, inborn errors in components of the IL-17 pathway have been associated with an increased susceptibility to fungal infections, first and foremost chronic mucocutaneous candidiasis (CMC). These include defects in the genes encoding IL-17F or IL-17RA [2]. Genome-wide association studies with patients suffering from CMC have identified additional polymorphisms that cause defects in the IL-17 response [2], clearly indicating that the IL-17 pathway plays a non-redundant role in antifungal immunity. This notion is supported by studies using animal models: mice deficient in IL-17 signaling or unable to mount robust IL-17 production are strongly impaired to control skin and mucosal infections with *Candida albicans* [3], [4]. In mice, IL-17 has also been shown to protect from other fungal infections including those caused by *Pneumocystis carinii* [5], *Histoplasmacapsulatum* [6], and *Aspergillus fumingatus* [7]. In line with its protective role at mucocutaneous surfaces, IL-17 acts mainly on epithelial cells of the skin and mucosal tissues. The cellular source of IL-17 however varies and depends on the inflammatory context and the site of infection.

## Which Cellular Sources Provide IL-17 during Fungal Infection?

CD4^+^ T cells are widely accepted as the major producers of IL-17 in response to fungal infection [8]. The differentiation of naïve T cells into Th17 effector cells is instructed by IL-6, which induces the essential transcriptional regulator RORγt, whereas IL-1β and IL-23 are involved in the maintenance of Th17 cells [8]. The cytokines necessary for Th17 differentiation are delivered mainly by antigen presenting cells, which recognize conserved fungal molecular patterns through C-type lectin receptors and Toll like receptors [8]. Differentiation of T cells into Th17 effector cells however is slow and takes up to a week. In contrast, the IL-17-dependent antifungal response is initiated within hours of initial pathogen encounter and the requirement for IL-17 to restrict fungal replication and dissemination manifests long before the Th17 developmental program is initiated, as studies in mouse models demonstrated [3], [4], [9], [10]. These findings clearly indicate the involvement of other cellular sources for IL-17. Subsets of γδ T cells and NKT cells as well as the more recently identified innate lymphoid cells (ILCs) act as important effectors of innate immunity providing IL-17 in response to specific stimuli [11]. As their name implies, ILCs are of lymphoid origin, but in contrast to lymphocytes they do not express RAG-dependent rearranged antigen-specific receptors [12]. Divided into three groups, ILCs mirror conventional T cell subsets based on their expression of specific transcription factors (Tbet, GATA3, RORγt) along with their ability to produce Th1, Th2, or Th17-associated cytokines [12]. IL-17 and IL-22 producing ILCs were termed ILC3 [12]. Similar to pathogenic Th17 cells, IL-17-secreting ILC3s have been implicated in the induction of colitis in mice and humans [13], [14]. In addition, ILC3s contribute to tissue repair and homeostasis [15]. Most importantly and in line with the host-protective function of IL-17 in the context of fungal infection, ILC3s have recently been implicated in the acute response to *C. albicans* [9]. Depletion of ILC3s renders mice highly susceptible to oropharyngeal candidiasis as does a deficiency in the IL-17 pathway [9]. In contrast, the lack of IL-22, another central ILC3 cytokine, only leads to a mild impairment in protection from oropharyngeal and cutaneous candidiasis [6], [7]. While ILC3s constitute a major source of IL-17 at mucosal surfaces, γδ T cells appear to be more important in the skin: they secrete high amounts of IL-17 during cutaneous candidiasis [16], albeit their contribution to protection from skin infection has not yet been formally proven. Finally, it remains to be demonstrated whether IL-17-dependent innate immunity to fungal pathogens other than *C. albicans* also involves γδ T cells and ILC3s.

## How Is IL-17 Production by ILCs Regulated?

In contrast to their adaptive counterparts that undergo a complicated developmental program to differentiate into T helper cell subsets upon immune activation, the functional commitment of innate IL-17 secreting cells is appointed during development. They constitutively express the lineage-specific transcription factor RORγt as well as receptors that allow them to promptly respond to cytokine stimulation induced upon microbial encounter. IL-23 and/or IL-1β, which are also involved in Th17 differentiation, act as key factors in the induction of innate IL-17 [11]. However, the relative role of each of these two cytokines appears to be context dependent. While innate IL-17 induction can occur independently of IL-23 in some cases [17], ILC3-derived IL-17 in the oral mucosa is strictly dependent on IL-23 and not affected by IL-1 receptor deficiency [9]. In the gut mucosa, IL-1β and IL-23 cooperate to induce IL-17 from ILC3s [18], and finally, IL-18 can replace IL-1β in combination with IL-23 to promote IL-17 secretion from γδ T cells [19]. Additional signals such as those mediated via pattern recognition receptors or NK cell receptors found on ILCs may also contribute to full activation [20], [21]. IL-17 secretion by ILC3s and γδ T cells may in fact be regulated by the integration of signals obtained from both cytokines and cell-bound receptors. Of interest in the context of fungal infections are γδ T cells expressing TLR1, TLR2, and Dectin-1, which were shown to produce IL-17 in response to PRR engagement and IL-23 stimulation, but independent of TCR signaling [22].

## From Mice to Man: Do ILCs Play a Role in Antifungal Immunity in Humans?

Most available data on ILCs comes from research with animal models. However, ILCs were also identified in humans [23]. Like their murine counterparts they reside in barrier tissues including the skin and the intestine, but they can also be found in the blood and secondary lymphoid organs, and they express RORγt and secrete IL-17 and IL-22 [23]. Although they likely contribute to the homeostatic balance with the intestinal microflora in healthy individuals, their accumulation in the intestine of IBD patients indicates a detrimental role of ILC3s in the pathogenesis of human inflammatory diseases [14]. Opportunistic fungal pathogens such as *Candida* ssp. reside as part of the human commensal microflora in the gastrointestinal tract and promote the formation of *Candida*-specific CD4^+^ T cell memory in steady state. This is in contrast to the murine intestinal flora, where the spectrum of *Candid*a spp. is more limited and subject to variation based on animal husbandry and sanitary conditions [24]. Accordingly, in humans the fungal load is generally thought to be controlled by the adaptive immune system. Frequently cited evidence for this is provided by HIV^+^ individuals who commonly manifest oropharyngeal candidiasis (thrush) when CD4^+^ T cell counts drop as a consequence of the viral infection. Surprisingly the loss of intestinal CD4^+^ cells during SIV infection in macaques—the simian equivalent of HIV in humans—is accompanied by the loss of intestinal IL-17-secreting ILC3s and the progression towards AIDS [25]. In another study, ILC3-derived cytokine production (but not the number of ILC3s) in the tonsils and buccal mucosa was found to be reduced during SIV infection [26]. Together this raises the question whether ILC numbers and/or effector functions are also affected in response to HIV in humans and thus may contribute to the predisposition of AIDS patients to fungal infection. Likewise, the genetic defects identified to cause congenital forms of CMC have all been associated with defects in adaptive IL-17 mediated immunity. Many of them however may also affect innate IL-17 production and a role of ILCs in the epidemiology of CMC should thus be considered.

## Do ILCs Impact on Long-term Protection?

Although ILCs are equipped for reacting promptly to acute microbial offense, they do not only impact the immediate response to infection but may also provide long-lasting effects with beneficial or detrimental consequences for the host. In fact, ILCs can limit the dissemination of commensals and thereby regulate tissue homeostasis, and they can promote tolerance to commensal bacteria by inhibiting adaptive T cell responses as animal experiments have recently shown [27], [28]. Conversely, ILCs have been implicated in chronic situations of intestinal inflammation in humans and mice and in the progression toward bacteria-induced colon cancer in a mouse model [13], [14], [29]. ILCs may also contribute to long-term protection from infections including those caused by opportunistic fungi, to which we are constantly exposed. The mechanisms, by which ILCs control sustained responses, remain to be defined. An intriguing possibility involves effects of ILCs on the adaptive immune system. Although no experimental evidence is available to date, such a scenario is supported by the observation that IL-17-secreting γδ T cells can promote Th17 responses [30], [31]. It is conceivable that ILCs could act similarly and thereby modulate quantitative and/or qualitative aspects of T cell differentiation. Future studies may provide evidence for this and if so, they should clarify whether innate IL-17 acts directly on T cells or whether it affects T cell differentiation by modulating the priming APCs. It is intriguing to speculate that the ILC-mediated effects could be linked to the recently proposed concept of trained innate immunity, where monocytes provide long-term protection from *C. albicans* [32].

## Concluding Remarks

IL-17 producing ILCs are important new players of the immune system, in particular in the host response to infectious agents, including fungal pathogens. Complementary to their function as immediate cytokine producers in response to infection, their function may also have far-reaching consequences. Future studies will provide a comprehensive understanding of ILCs as effectors as well as modulators of the immune system.
